# Plasma Extracellular Vesicles Play a Role in Immune System Modulation in Minimal Hepatic Encephalopathy

**DOI:** 10.3390/ijms232012335

**Published:** 2022-10-15

**Authors:** Juan José Gallego, Alessandra Fiorillo, Franc Casanova-Ferrer, Amparo Urios, María-Pilar Ballester, Lucia Durbán, Javier Megías, Teresa Rubio, Andrea Cabrera-Pastor, Desamparados Escudero-García, Vicente Felipo, Carmina Montoliu

**Affiliations:** 1 Fundación de Investigación Hospital Clínico Universitario de Valencia-INCLIVA, 46010 Valencia, Spain; 2 Servicio de Medicina Digestiva, Hospital Clínico Universitario de Valencia, 46010 Valencia, Spain; 3 Servicio de Medicina Digestiva, Hospital Arnau de Vilanova, 46015 Valencia, Spain; 4 Departamento de Patología, Universidad de Valencia, 46010 Valencia, Spain; 5 Laboratory of Neurobiology, Centro Investigación Príncipe Felipe, 46012 Valencia, Spain

**Keywords:** minimal hepatic encephalopathy, extracellular vesicles, miRNAs, CD4+ T lymphocytes

## Abstract

Minimal hepatic encephalopathy (MHE) is associated with changes in the immune system including an increased pro-inflammatory environment and altered differentiation of CD4+ T lymphocytes. The mechanisms remain unknown. Changes in extracellular vesicle (EV) cargo including proteins and miRNAs could play a main role as mediators of immune system changes associated with MHE. The aim was to assess whether plasma EVs from MHE patients played a role in inducing the pro-inflammatory environment and altered differentiation of CD4+ T lymphocyte subtypes in MHE patients. We characterized the miRNA and protein cargo of plasma EVs from 50 cirrhotic patients (27 without and 23 with MHE) and 24 controls. CD4+ T cells from the controls were cultured with plasma EVs from the three groups of study, and the cytokine release and differentiation to CD4+ T-cell subtypes were assessed. Plasma EVs from MHE patients had altered miRNA and protein contents, and were enriched in inflammatory factors compared to the controls and patients without MHE. EVs from MHE patients modulated the expression of pro-inflammatory IL-17, IL-21, and TNF-α and anti-inflammatory TGF-β in cultured CD4+ T lymphocytes, and increased the proportion of Th follicular and Treg cells and the activation of Th17 cells. In conclusion, plasma EVs could play an important role in the induction of immune changes observed in MHE.

## 1. Introduction

Around 30–50% of cirrhotic patients show minimal hepatic encephalopathy (MHE) with attention deficits, psychomotor slowing, and mild cognitive impairment, which reduce the quality of life and life span [1,2,3]. The mechanisms leading to MHE involve the synergistic contribution of hyperammonemia and inflammation [4]. Patients with MHE show a more pro-inflammatory microenvironment in blood, with high levels of pro-inflammatory cytokines such as IL-21, IL-17, TNF-α and CCL20, leading to a cascade of immune responses that are finally transduced to the brain, resulting in the cognitive and motor impairment associated with MHE [5]. In blood, naive CD4+ T lymphocytes may differentiate into different effector T helper (Th) and regulatory (Treg) cell subsets: Th1, Th2, Th17, Th22, Th follicular (Tfh), or iTreg [6]. Each subset of CD4+ T cells are characterized by the transcription factors they express and the cytokines they release. Dysregulation of this differentiation results in the pathogenesis of different autoimmune and inflammatory diseases [7]. MHE is associated with the increased activation of CD4+ T lymphocytes and altered differentiation to Tfh and Th22, with increased expression of the transcription factors BCL6 and AHR [5]. The mechanisms by which these changes in the immune system are induced in MHE are unknown. Extracellular vesicles (EVs) play an important role in the modulation of the immune system, emerging as key players in shaping the immune responses due to their ability to modulate the microenvironment in the immune system [8,9]. EVs are membranous vesicles released by most cells [10]. The composition of EVs may change according to the tissue and cell type of origin as well as their physiological status. Once captured by the target cells, EVs can release their contents into the cytosol, inducing biological effects that can also be mediated by the action of the EV surface molecules [10]. The cargo of EVs such as proteins and miRNAs play a main role in the mediation of immune and inflammatory responses, and in diseases with a significant inflammatory component [11]. Plasma EVs from hyperammonemic rats, an animal model of MHE, carry the molecules that are necessary and sufficient to trigger neuroinflammation in the cerebellum and the mechanisms leading to motor incoordination in normal rats [12].

Depending on their cellular source, EVs carry at their surface a plethora of membrane-associated molecules that can modulate the response of immune cells including the T-cell receptor (TCRs), major histocompatibility complex (MHC)-associated peptide antigen, costimulatory, and inhibitory receptors. In autoimmune diseases, EVs modulate the differentiation, function, and stability of Th subsets and affect the ability of Tregs to control effector T cells [9].

The main aim of this work was to assess whether EVs isolated from the plasma of patients with MHE play a role in the induction of the pro-inflammatory environment and the altered differentiation of CD4+ T lymphocyte subtypes in patients with MHE [5]. We isolated and characterized the plasma EVs from the controls and cirrhotic patients without or with MHE, and performed a proteomic and miRNA analysis to assess whether there were differences in the cargo of the plasma EVs isolated. We also analyzed different markers of immune system cells and CNS cells to shed light on the cell type originating the plasma EVs, and the content of molecules involved in immune system activation, the pro-inflammatory environment, and ammonia metabolism. Finally, we assessed whether the addition of plasma EVs (from the controls and patients without and with MHE) to CD4+ T cell cultures from the control subjects induced changes in the interleukins released and in their differentiation to CD4+ T cell subtypes.

## 2. Results

### 2.1. Cirrhotic Patients Show Lower Number of Plasma EVs which Show Increased Size

**Figure 1:**
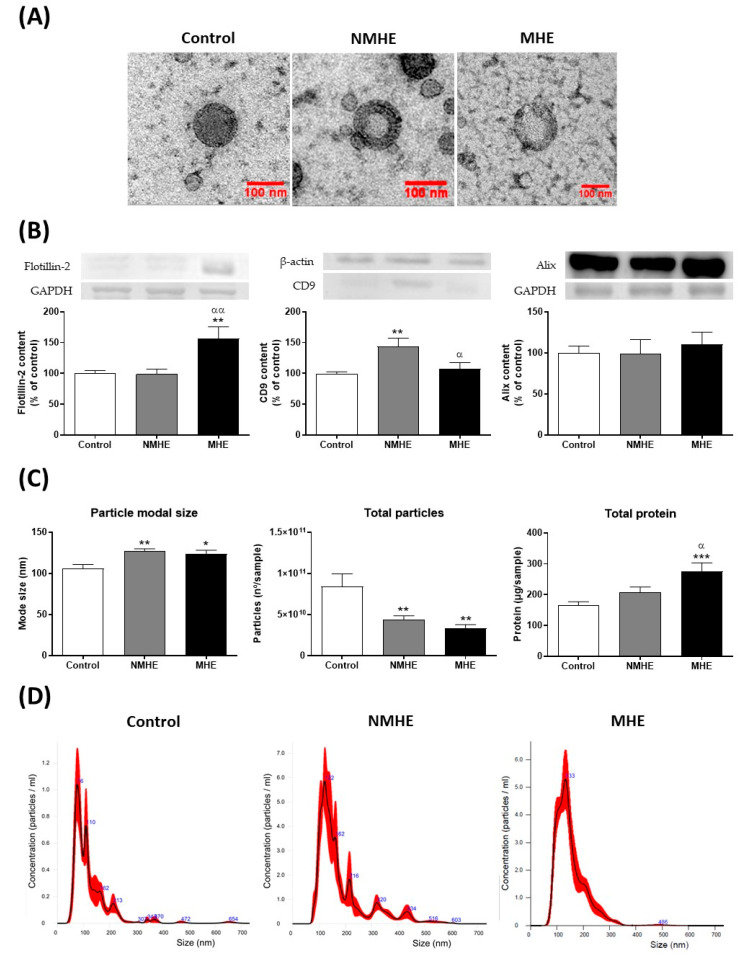
Characterization of extracellular vesicles isolated from the plasma of the control and patients without and with MHE. (**A**) Representative images obtained by transmission electron microscopy after negative staining. (**B**) Content of markers Flotillin-2, CD9, and Alix analyzed by Western blot; β-actin or GAPDH were used as the loading controls. (**C**) Particle size mode (nm), total particles per sample measured by nanoparticle tracking analysis, and total protein (µg/sample) in EVs. The total particles and total protein of EVs were referred to the initial volume of plasma used to isolate EVs (5 mL), the same for all samples. Values are the mean ± SEM of 10–25 samples per group. Data were analyzed by univariate analysis of covariance (ANCOVA) with age included as a covariate, followed by the Tukey post hoc test. (**D**) Example of the particle size distribution profiles measured by NTA in the three groups of study. NMHE, MHE, patients without and with minimal hepatic encephalopathy, respectively. Values significantly different from the control are indicated by an asterisk (*) and from NMHE patients by α (*/α: *p* < 0.05; **/αα: *p* < 0.01; ***: *p* < 0.001).

Figure 1A shows the representative images obtained by transmission electron microscopy for extracellular vesicles isolated from the plasma of the control and patients without and with MHE. EV markers such as Flotillin-2, CD9, and Alix are shown in Figure 1B. As shown in Figure 1C, the amount of EVs was reduced (*p* < 0.01) in both patient groups compared to the controls. The size distribution profile was analyzed by nanoparticle tracking analysis (Figure 1D). The average particle size (mode) of the EVs was higher in patients than in the controls, and the total protein of EVs was increased in patients with MHE (276 ± 28 µg) compared to the patients without MHE (209 ± 17 µg; *p* < 0.05) and the controls (167 ± 11 µg; *p* < 0.001) (Figure 1C).

### 2.2. MHE Alters Protein and miRNAs Cargo of EVs in Plasma

**Figure 2:**
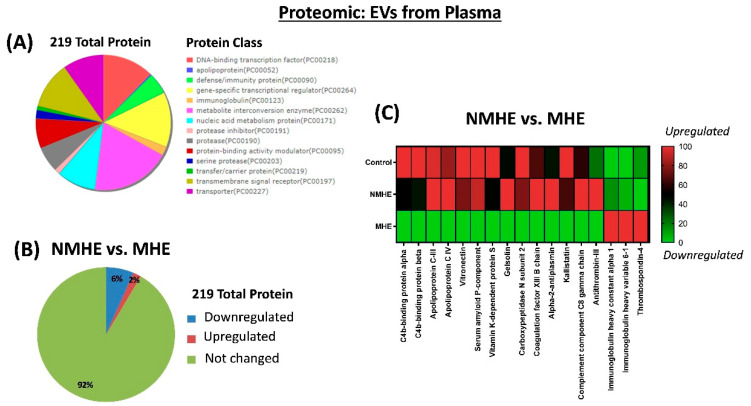
Proteomic profiling of EVs purified from plasma and differently expressed in patients with MHE. (**A**) Gene Ontology (GO) analysis to determine the protein class for the 219 proteins identified. (**B**) Percentage of the upregulated and downregulated proteins in MHE. (**C**) Heat-map of proteins differently expressed in EVs from patients with MHE and obtained by SWATH analysis as indicated in the methods. NMHE, MHE, patients without and with minimal hepatic encephalopathy, respectively.

We analyzed the protein cargo by performing a proteomics analysis, which analysis identified 219 proteins. Gene Ontology (GO) analysis was applied to these 219 proteins to assess which pathways were more affected in the MHE patients. After using the Uniprot mammalia library with false discovery rate (FDR) identification and the analysis of individual SWATH experiments, the 219 proteins (FDR < 1%) were quantified in the 50 samples. Proteomic profiling using the Gene Ontology (GO) platform showed that the proteins were classified in the following: DNA-binding transcription factor, apolipoprotein, defense/immunity protein, gene-specific transcriptional regulator, immunoglobulin, metabolite interconversion enzyme, nucleic acid metabolism protein, protease inhibitor, protease, protein-binding activity modulator, serine protease, transfer/carrier protein, transmembrane signal receptor and transporter (Figure 2A).

Differential expression analysis identified 17 proteins whose contents were different in EVs from MHE and NMHE patients. Fourteen proteins (6%) were downregulated, three proteins (2%) were upregulated and 202 proteins (92%) remained unaltered (Figure 2B). The proteins differentially expressed in MHE are shown in Figure 2C.

To gain insights into the potential biological function of proteins differentially expressed, we applied Gene Ontology (GO) analysis (Table S2). The differences in EV proteins are mainly associated with biological processes of the regulation of opsonization, response to symbiotic bacterium, negative regulation of complement activation (classical pathway), very low density lipoprotein particle assembly, negative regulation of viral entry into the host cell, acute inflammatory response, complement activation (classical pathway), renal system process, platelet degranulation, phagocytosis, negative regulation of endopeptidase activity, blood coagulation, and innate immune response (Table S2).

**Figure 3:**
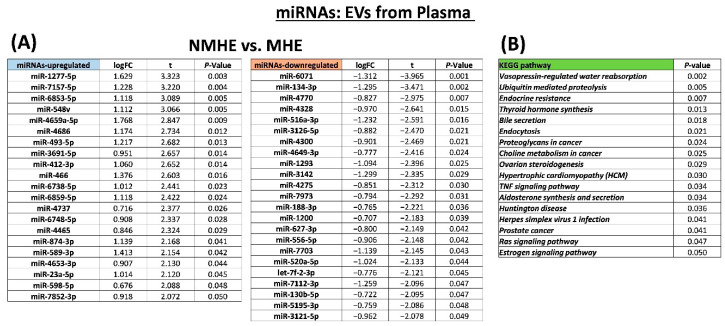
The miRNAs of EVs purified from plasma and differently expressed in patients with MHE. (**A**) List of the upregulated and downregulated miRNAs in EVs from MHE and obtained as indicated in the methods. (**B**) The KEGG pathways obtained from the target genes of miRNAs with differential expression in MHE as indicated in the methods. NMHE, MHE, patients without and with minimal hepatic encephalopathy, respectively.

We also analyzed the miRNA cargo using the HTG EdgeSeq System, which quantified the expression of 2083 miRNAs. After applying the normalization method CQN to remove the GC-content bias and correct for global distortions in the HTSeq data, linear modeling from the limma R/Bioconductor package was performed to find the differentially expressed miRNAs. The expression of 44 miRNAs was different in EVs from the MHE and NMHE patients. Twenty-one miRNAs were upregulated and 23 miRNAs were downregulated (Figure 3A).

To gain insights into the potential biological function of miRNAs differentially expressed in MHE, the target genes of miRNAs were found by screening five different databases (miRDB, miRTar, PITA, miRWalk, and TargetScan), keeping those pairs that were annotated in at least three databases and belonging to miRWalk and TargetScan. Differential expression information from miRNAs to the target genes was transferred to the KEGG pathway database annotation and 17 pathways showed significant relevance (Figure 3B). Some of these pathways are associated with biological processes such as vasopressin-regulated water reabsorption, ubiquitin mediated proteolysis, endocrine resistance, or the TNF signaling pathway.

### 2.3. EVs from Plasma of Patients with MHE Show Altered Content of Immune and Neuronal Cells Markers

To assess the cell origin of the EVs we analyzed by Western blot different markers: CD3 (for lymphocytes), CD4 (for T helper lymphocytes), CD8 (for T cytotoxic lymphocytes), CD19 (for B lymphocytes), CD86 (for antigen presenting cells), CD14 (for monocytes), and CD16 (for pro-inflammatory monocytes). We also assessed the expression of two markers related to CNS cells: L1CAM (neurons) and CD13 (microglia).

**Figure 4:**
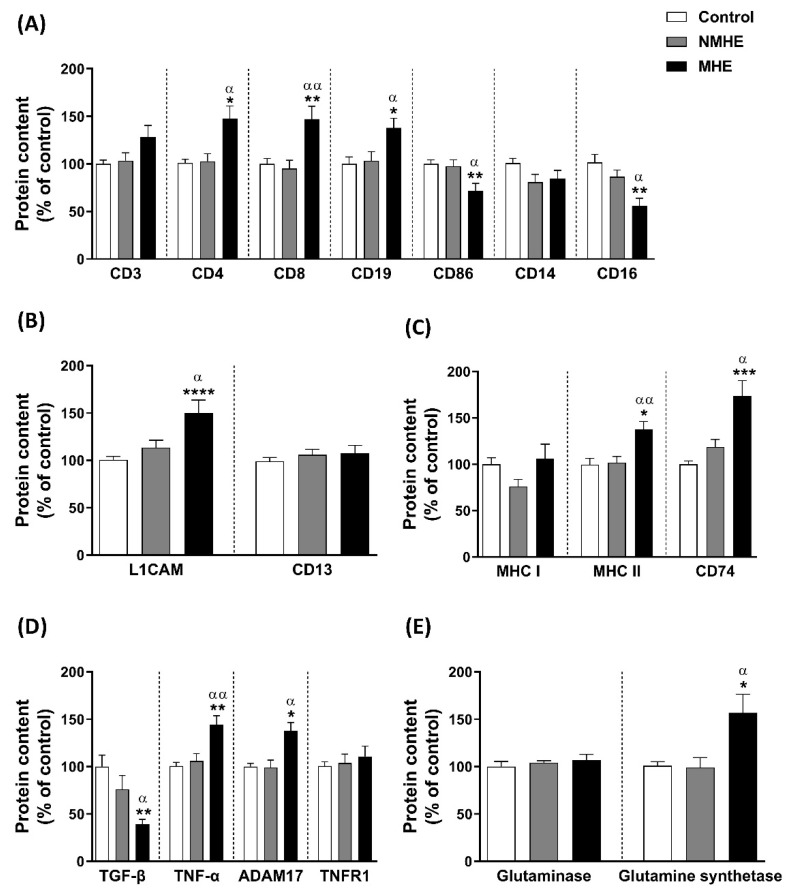
Extracellular vesicle protein cargo. Content of (**A**) markers related to the immunity cells CD3, CD4, CD8, CD19, CD86, CD14 and CD16, (**B**) markers related to the CNS cells L1CAM and CD13, (**C**) molecules implicated in the activation of the immune system and presenting antigens to T cells MHC I, MHC II, and CD74, (**D**) molecules related to the inflammation environment TGF-β, TNF-α, ADAM17, and TNFR1, and (**E**) enzymes related to ammonia metabolism, glutaminase, and glutamine synthetase. Values are expressed as the percentage of the control and are the mean ± SEM. Data were analyzed by univariate analysis of covariance (ANCOVA) with age included as a covariate, followed by the Tukey post hoc test. NMHE, MHE, patients without and with minimal hepatic encephalopathy, respectively. Values significantly different from the control are indicated by an asterisk (*) and from NMHE patients by α (*/α: *p* < 0.05; **/αα: *p* < 0.01; ***: *p* < 0.001; ****: *p* < 0.0001).

EVs from patients with MHE showed increased levels of CD4 (148 ± 13%; *p* < 0.05), CD8 (147 ± 14%; *p* < 0.01), and CD19 (138 ± 10%; *p* < 0.05) compared to the controls and to NMHE (Figure 4A). EVs from MHE patients showed decreased levels of CD16 (56 ± 8%; *p* < 0.01) and CD86 (72 ± 8%; *p* < 0.01) compared to the controls and NMHE patients (*p* < 0.05) (Figure 4A). The levels of CD3 and CD14 did not change between groups (Figure 4A).

EVs from the plasma of MHE patients were enriched in L1CAM (150 ± 14%) compared to the controls (*p* < 0.0001) and NMHE patients (*p* < 0.05), suggesting an increase in EVs from neuronal origin (Figure 4B). No changes in the content of the microglia marker CD13 were observed (Figure 4B).

### 2.4. Content of Presenting Antigen Molecules in Plasma EVs

The EVs from patients with MHE showed an increased content of MHC II (138 ± 9%) and CD74 (174 ± 17%) compared to the controls (*p* < 0.05 and *p* < 0.001, respectively) and to NMHE (*p* < 0.01 and *p* < 0.05, respectively). The levels of MHC I did not change between groups (Figure 4C).

### 2.5. EVs from Plasma of MHE Patients Show an Increase in Pro-Inflammatory Molecules and a Reduction in Anti-Inflammatory Molecules

Plasma EVs from MHE patients showed a significant increase in inflammatory markers such as TNF-α (144 ± 10%) and ADAM17 (138 ± 9%) compared to the controls (*p* < 0.01 and *p* < 0.05, respectively) and NMHE patients (*p* < 0.01 and *p* < 0.05, respectively) (Figure 4D). There were no changes in the TNFR1 receptor, and there was a significant decrease in the anti-inflammatory cytokine TGF-β (39 ± 5%) (MHE vs. controls: *p* < 0.01; MHE vs NMHE: *p* < 0.05) (Figure 4D).

We correlated the inflammatory markers in EVs with the PHES and with the activated lymphocyte populations. There was a significant negative correlation between the TNF-α content in EVs, and a positive correlation of TGF-β with the PHES (r = *−*0.419; *p* < 0.01, and r = 0.297; *p* < 0.05, respectively) (Table S3). Moreover, we found a significant positive correlation (r = 0.470; *p* = 0.015) between the TNF-α in EVs and the activation of memory CD4+ T cells, and a negative correlation between TGF-β and these activated T cells (r = *−*0.462; *p* = 0.02) (Table S3).

### 2.6. Ammonia Metabolism Enzyme Content in Plasma EVs

We analyzed the content of two enzymes involved in ammonia metabolism: glutaminase and glutamine synthetase. EVs from the plasma MHE showed an increase in the glutamine synthetase content (157 ± 19%) compared to the controls and NMHE groups (*p* < 0.05). The content of glutaminase was not altered (Figure 4E).

### 2.7. EVs from Plasma of MHE Patients Modulate the Expression of Pro-Inflammatory Cytokines IL-17, IL-21, and TNF-α, and the Anti-Inflammatory Cytokine TGF-β in Cultured CD4+ T Lymphocytes

**Figure 5:**
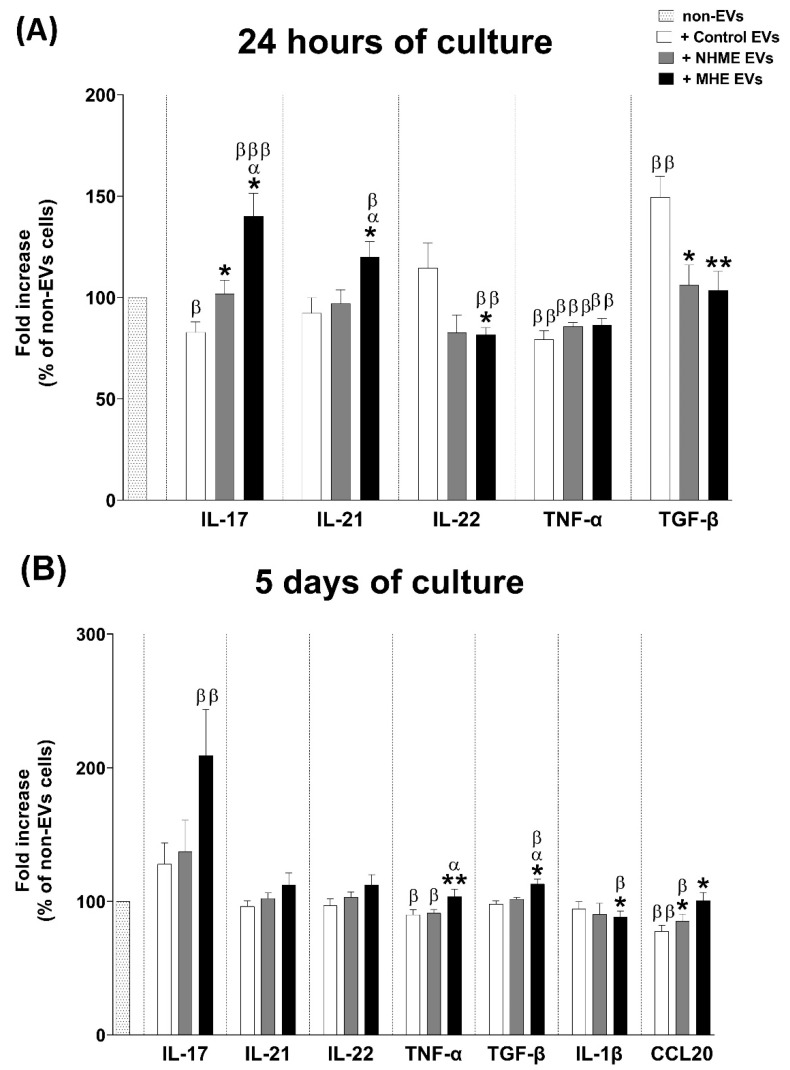
Cytokine release by CD4+ T cells after 24 h and 5 days cultured with human plasma EVs. (**A**) After 24 h of CD4+ T cells cultured with plasma EVs from the control (control EVs), without (NMHE EVs) or with minimal hepatic encephalopathy (MHE EVs) subjects, the levels of IL-17, IL-21, IL-22, TGF-β, and TNF-α were measured in the supernatant by ELISA. (**B**) Levels of IL-17, IL-21, IL-22, TGF-β, TNF-α, IL-1β, and CCL20 measured after 5 days of culture. A condition without EV treatment was also included as a reference (non-EVS). Values are given as the fold increase of the cytokine levels over the non-EV condition, which was considered as 1. Values are the mean ± SEM. Eight independent experiments were conducted for all conditions. Values significantly different from the control EV condition are indicated by asterisks (*), from the NMHE EV condition by α, and from the non-EV condition by β (*/α/β: *p* < 0.05; **/ββ: *p* < 0.01; βββ: *p* < 0.001).

To evaluate the effects of plasma EVs on the profile of the cytokines released by CD4+ T cultures, we measured the IL-17, IL-21, IL-22, TNF-α, and TGF-β levels at 24 h of culture. Incubation of the CD4+ control cells with EVs from MHE patients induced a significant increase in the IL-17 and IL-21 levels at 24 h (140 ± 11% and 120 ± 8%, respectively), compared to the condition without the addition of EVs (non-EVs) (*p* < 0.001 for IL-17; *p* < 0.05 for IL-21) or with the addition of EVs from the controls (*p* < 0.05 for both interleukins) or NMHE patients (*p* < 0.05 for both interleukins) (Figure 5A). In contrast, the IL-22 levels decreased after the addition of EVs from MHE patients (82 ± 5%) compared to the controls (*p* < 0.05) and to the non-EV condition (*p* < 0.01) (Figure 5A). The TNF-α levels were significantly decreased by the addition of EVs from the controls or patients (Figure 5A). EVs from the controls reduced the expression of IL-17 (83 ± 5%) and induced a significant release of TGF-β at 24 h (150 ± 10%) compared to the non-EV condition and with the addition of EVs from patients (Figure 5A). The levels of cytokines IL-21 and IL-22 were not affected. EVs from the NMHE patients did not affect the expression of IL-17, IL-21, IL-22, and TGF-β.

After 5 days of culture, the IL-17 levels remained increased (209 ± 34%) (Figure 5B) and there were also significant increases in the TGF-β (113 ± 4%) levels in the presence of EVs from MHE compared to the EVs from the controls and NMHE patients (Figure 5B).

In the presence of EVs from MHE patients, the IL1-β levels were lower than the non-EV condition and control EVs after 5 days of culture (Figure 5B).

There was a significant decrease in TNF-α and CCL20 after 5 days of culture after the addition of EVs from the controls and NMHE patients compared to the non-EV condition. The levels of these cytokines did not change in the presence of EVs from MHE patients, being significantly higher than those from the controls (*p* < 0.01 for TNF-α; *p* < 0.05 for CCL20) and from NMHE (*p* < 0.05 for TNF-α) (Figure 5B).

### 2.8. EVs from Plasma of MHE Patients Increased the Proportion of Th Follicular and Treg Cells and Activation of Th17 Cells

We assessed whether the addition of EVs induced the differentiation of CD4+ T lymphocytes to different cell subtypes. After 5 days of the addition of EVs, the expression levels of the transcription factors TBX21, GATA3, RORC, BCL6, AHR, and FOXP3 were analyzed by qPCR as transcription factors selective for Th1, Th2, Th17, Th follicular, Th22, and Tregs, respectively.

**Figure 6:**
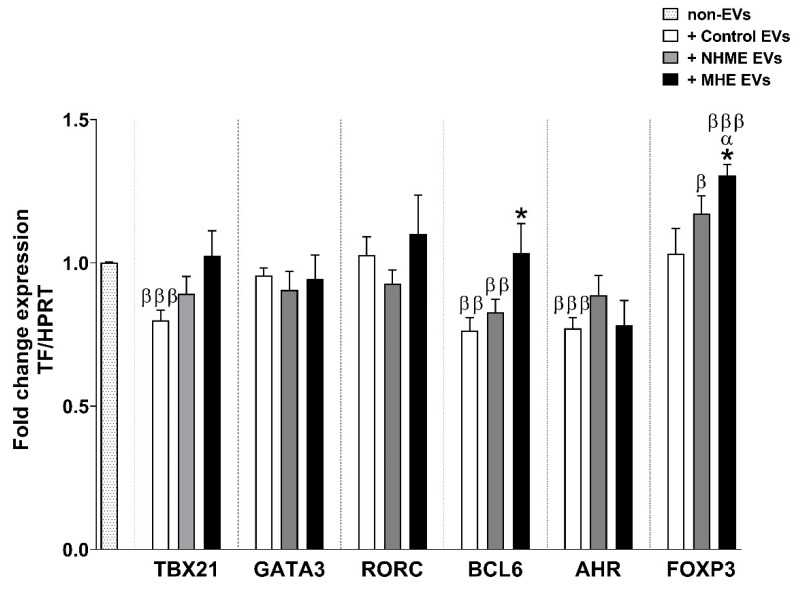
Transcription factor expression in CD4+ T cells after 5 days of treatment with human plasma EVs. After 5 days of CD4+ T cells cultured with plasma EVs from the control (control EVs), without (NMHE EVs) or with minimal hepatic encephalopathy (MHE EVs) subjects, the expression of TBX21, GATA3, RORC, BCL6, AHR, and FOXP3 were quantified by qPCR. A condition without exosome treatment was also included as a reference (non-EVs). Values are given as the normalized target gene amount relative to non-EV conditions, which were considered as 1. Values are the mean ± SEM. Eight independent experiments were conducted for all conditions. Values significantly different from the control EV condition are indicated by asterisks (*), from the NMHE EV condition by α and from the non-EV condition by β (*/α/β: *p* < 0.05; ββ: *p* < 0.01; βββ: *p* < 0.001).

Cultures with the control EVs led to a reduction in TBX21 and AHR expression in the CD4+ T cells compared with the non-EV condition (0.80 ± 0.03 and 0.77 ± 0.04, respectively; *p* < 0.001), while its expression was not affected by the NMHE or MHE EVs (Figure 6). GATA3 and RORC expression were not affected by any kind of EVs added (Figure 6). The expression of BCL6, a marker for Th follicular cells, was significantly increased (*p* < 0.05) after 5 days of incubation with EVs from MHE patients compared to those from the control subjects. In contrast, the expression of this transcription factor was reduced in the presence of EVs from the controls and NMHE patients with respect to the condition without EVs (*p* < 0.01) (Figure 6).

In cells treated with EVs from MHE patients, the expression of FOXP3 was significantly increased compared to the other conditions (1.31 ± 0.04; *p* < 0.001 vs. the non-EV condition; *p* < 0.05 vs. the control and NMHE EVs). EVs from NMHE patients also induced a higher expression of FOXP3 compared with the non-EV condition (*p* < 0.05) (Figure 6). The enhanced FOXP3 expression, together with the increased TGF-β levels (Figure 5B), suggest that MHE EVs could lead CD4+ T cells to differentiate into Treg cells.

Although the expression of the transcription factor RORC did not change in the presence of the EVs of MHE patients, suggesting that they did not increase the differentiation of CD4+ T cells to Th17, the increased levels of IL-17 and CCL20 at 5 days of culture would indicate that EVs from MHE patients would induce the activation of Th17 cells in the cultures.

## 3. Discussion

We showed that plasma EVs from MHE patients had altered miRNA and protein contents and were enriched in inflammatory factors, which could play a main role in the changes in peripheral inflammation described in these patients. Moreover, EVs from MHE patients showed altered contents of immune and neuronal cell markers, indicating a different cellular origin than for EVs from NMHE patients and the controls.

We isolated and characterized the plasma EVs from the controls and patients, and we found a decrease in the total number of EVs and an increase in their size in plasma from cirrhotic patients. EVs are released from all kinds of cells in blood, but tend to be enriched in EVs derived from platelets, monocytes, and dendritic cells in healthy subjects [13]. Platelet-derived EVs account for 2/3 of circulating EVs in the blood [13]. Cirrhotic patients generally present a significant thrombocytopenia compared to the healthy controls, which could explain the reduction in the number of EVs in patients.

The reduction in total particles, similar to cirrhotic patients, was observed in other inflammatory pathologies such as HIV [14]. The increase in the size of the EVs was also detected in HIV patients compared to the controls, and correlated with immunological parameters reflecting disease progression [15].

We previously found an increase in the intermediate monocyte population in plasma from MHE patients [5] and we expected to also find an increase in the CD16 marker in EVs, but this was not the case. As far as we know, there have not been any studies characterizing the CD16^+^ EV content in MHE patients. An inverse association has been reported between membrane proteins CD14^+^ and CD16^+^ EVs and liver fibrosis severity in NAFLD [16]. According to this, both groups of cirrhotic patients should show a decrease in both CD14 and CD16 markers in EVs compared to the control EVs. There was a trend to decrease in both groups in the case of CD14, and for CD16, the decrease was higher and significant in the MHE group, indicating that this decrease could be associated with MHE. The EVs enriched in inflammatory markers could be derived from other cell types such as autoreactive CD4+ CD28 T lymphocytes, which are increased in MHE patients [5]. This cellular type produces large amounts of TNFa and IFNg, and promotes a pro-inflammatory environment [17]. Moreover, the persistent activation of CD4+ T cells found in MHE patients [5] could contribute to altered patterns of cytokines and CD4+ T cell differentiation. These activated populations could be delivering a pool of EVs containing pro-inflammatory factors, thus promoting a pro-inflammatory environment in MHE patients.

Plasma from patients with MHE was enriched in EVs from neurons (L1CAM), T helper lymphocytes (CD4), T cytotoxic lymphocytes (CD8), and B lymphocytes (CD19) compared to those from the other studied groups. This enrichment could reveal a dysregulation in these cell types. Some studies have reported an increase in circulating EVs that was related to activation, metabolic stress, inflammatory signals, or oxidative stress [18,19]. It is noteworthy that the activation of CD4, CD8, and B lymphocytes was increased in patients with MHE, as indicated by the increased content of the activation marker CD69 [5]. The increased content of CD4, CD8, and CD19 in EVs from MHE patients would be a consequence of the increased release of EVs by these subtypes of lymphocytes as a consequence of the increased activation. However, we cannot rule out that the observed increases could be due not only to a higher number of EVs released by these cell types, but also to an upregulation of the respective markers.

Concerning neuron derived EVs, Lachenal et al. [20] demonstrated that EV release was regulated by glutamatergic activity, which is altered in animal models of MHE [21,22]. The secretion of neuron derived EVs is modulated by synaptic AMPA- and NMDA-receptors [20]. The increase in L1CAM content in EVs from the plasma of MHE patients would indicate an increase in neuron derived EV secretion, which could be due to a tonic activation of NMDA receptors induced by hyperammonemia [22].

It has been proposed that the analysis of the content of neuron-derived exosomes isolated from blood may serve to predict and diagnose cognitive impairment associated with neurodegenerative diseases such as Alzheimer’s disease or HIV [14,23].

Future studies analyzing the content of EVs with a neuronal origin isolated from the blood of patients with MHE may identify biomarkers for neuronal alterations in the brain and for cognitive impairment, which may be useful to diagnose MHE in cirrhotic patients.

EVs from the plasma of MHE patients promoted the activation of pro-inflammatory cytokine pathways, leading to an increased differentiation to Th follicular and Tregs cells, and to the activation of Th17 lymphocytes in cultured CD4+ T lymphocytes from the control subjects.

The profile of interleukins released by CD4+ T lymphocytes in culture can be modified by the EVs in several ways: (1) proteins that act within the cell upon fusion of the EV; (2) proteins in EVs that act on the cell surface, for example, the MHC II (increase in EVs from MHE), which could be activating CD4+ T lymphocytes; (3) miRNAs that modulate the translation or stabilize/destabilize transcription factors; or (4) proteins in the surface of EVs (e.g., TNF-α) may activate receptors in CD4+ T lymphocytes.

The analysis of differentially expressed proteins shows that EVs from the MHE patients included eight downregulated and one upregulated proteins, which are common to those found in EVs from hyperammonemic rats [12]. One downregulated protein is gelsolin, whose plasma concentration is decreased in inflammatory diseases as well as in neurological disorders [24].

EVs from MHE patients had a higher TNF-α and ADAM17 content than the EVs from the controls and patients without MHE, a tendency to increase TNFR1, and a lower content of TGF-β. When added to T CD4+ lymphocyte cultures, high TNF-α and low TGF-β in the EVs would favor a pro-inflammatory environment.

One of the processes relating miRNA cargo differences between NMHE and MHE patients is the TNF signaling pathway. It has been described that TNF-α is a direct target of miR-130b [25]. Mechanistic analyses indicated that tumor necrosis factor-α (TNF-α) was regulated by miR-130b directly. MiR-130b attenuated nuclear factor-κB (NF-κB) signaling and its downstream gene vascular endothelial growth factor-A (VEGFA) by directly inhibiting TNF-α expression. Accordingly, we found a downregulation of mir-130b and an increase in TNF-α in the EVs from MHE patients.

TNF-α can act on the target cell bound to the EVs or may be cut by ADAM17 to its soluble form, which is more bioactive; moreover, the fusion of the extracellular vesicles to the target cell would keep the TNFR1 stable, making the cell more susceptible to the action of TNF-α. TNF-α in EVs derived from dendritic cells may activate endothelial cells and their inflammatory pathways via NFκβ [26]. Furthermore, EVs derived from Crohn’s macrophages participate in activating the TNFR2-TRAF2-NFκβ pathway in CD4+ T lymphocytes [27]. Zhang et al. [28] showed that EVs derived from fibroblasts from patients with rheumatoid arthritis contained TNF-α, maintained the proliferation of CD4+ T lymphocytes, and induced the production of IL-2 and IFN-γ. They also found that TNF-α associated with EVs increased the transcription mediated by the NFκβ and Akt pathways, which was associated with the increased synthesis of pro-inflammatory cytokines [28]. Much like in these studies, the TNF-α in EVs from the MHE patients could activate receptors on CD4+ T lymphocytes, which would lead to the activation of the NFκβ pathway and the increase in pro-inflammatory cytokine production observed at 24 h and 5 days of culture.

We previously showed an increase in the activation of all subtypes of CD4^+^ lymphocytes in MHE [5]. In this study, we found a significant positive correlation between the TNF-α in EVs, the activation of memory CD4^+^ T cells, and a negative correlation between TGF-β from EVs and these activated T-cells, which would enhance the pro-inflammatory environment found in MHE patients. The enhanced content of TNF-α in EVs from MHE patients would contribute to activating CD4 lymphocytes, which, in turn, would trigger MHE, as reported by Mangas-Losada et al. [5].

Both dendritic cells and B lymphocytes express MHC II on their surface, and in the membrane of their EVs. Since the dendritic cell marker (CD86) is decreased in MHE EVs, the increased content of MHC II in EVs from MHE patients would come from the EVs from B lymphocytes. B lymphocytes degrade 50% of the peptide–MHC II complex intracellularly, but part of the vesicles whose destiny it is to fuse to lysosomes escape and are released in the form of EVs. This EV-associated peptide–MHC II complex is capable of stimulating cytokine production in CD4 T cells with a low activation threshold [29,30]. The increased content of MHC II in EVs from MHE patients may also contribute to the activation of CD4 T lymphocytes.

EVs from the control subjects induced a decrease in the transcription factors characteristic of Th1, Th follicular, and Th22 (TBX21, BCL6, and AHR, respectively) observed at 5 days of incubation. This could be mediated by TGF-β, which had a higher content in the control EVs than in the EVs from the NMHE and MHE patients. Álvarez et al. [31] showed that TGF-β contained in EVs derived from endometrial mesenchymal stem cells inhibited the generation of effector phenotypes in cultures of CD4+ T lymphocytes. In these experiments, the stimulation of CD4+ T lymphocytes without EVs generated effector phenotypes, while this effect was inhibited in cells co-cultured with EVs derived from endometrial mesenchymal stem cells. The inhibition in the generation of effector phenotypes in CD4+ T lymphocytes was reversed by adding a TGF-β blocker, confirming that TGF-β mediates the inhibition in the generation of effector phenotypes in CD4+ T lymphocytes.

The higher content of TGF-β in EVs mediates the decrease in Th1, Th follicular, and Th22 phenotypes in cultures incubated with EVs from the control subjects and of follicular Th phenotypes in cultures incubated with EVs from subjects without MHE. The low content of TGF-β in the EVs from patients with MHE would not inhibit the generation of effector phenotypes in cultures of CD4+ T lymphocytes, allowing the polarization of these cells to be influenced by the other components (TNF-α, MHC II,…) present in the EVs of MHE.

The miRNAs present in EVs may affect specific pathways, altering the expression of cytokines and transcription factors. One of these pathways would be the PI3K-AKT pathway. AKT activates FOXO1 and FOXO3, which travel to the nucleus and activate FOXP3 transcription, essential for Tregs development and function. A stable balance must be maintained in AKT activation, since low or excessive AKT activation/phosphorylation negatively affects Tregs [32,33]. miR-4465 in EVs inhibits PTEN in pancreatic cancer cells, enhancing the activation/phosphorylation of AKT [34]. miR-4465 was increased in EVs from MHE and could inhibit PTEN expression, leading to greater activation/phosphorylation of AKT [35].

On the other hand, miRNAs from EVs could play a role in the induction of MHE. We found that mir-130b-5p was downregulated in MHE. This miRNA is associated with cognitive impairment. The expression level of miR-130b is downregulated in patients with vascular dementia and in Alzheimer’s disease [36]. This miRNA is also downregulated in the hippocampal tissue of rats with diabetic encephalopathy, and the overexpression of this miRNA attenuated cognitive impairment and hippocampal damage in this rat model [37].

miR-130b-5p is underexpressed in EVs from MHE; this miRNA inhibits the TLR4-NFkβ pathway in microglial cultures, reducing associated inflammation [38].

The lower expression of miR-130b-5p found in EVs from MHE patients would result in a lack of inhibition of the production of pro-inflammatory cytokines. On the other hand, the addition of EVs from the controls or patients without MHE, enriched in miRNA-130-5p, would reduce the expression of pro-inflammatory cytokines in CD4 T cell cultures, as was the case for the expression of IL-17, TNF-α, and CCL20 in cultures at 5 days.

Another miRNA, differentially expressed in MHE patients was let-7f, which was downregulated in the EVs from MHE patients. This miRNA suppresses Th17 differentiation via targeting STAT3 in multiple sclerosis [39]. The reduced content of let-7f in MHE patients may contribute to the increased differentiation of CD4 lymphocytes to Th17 reported in MHE patients [5] and in the induction of the pro-inflammatory environment associated with MHE.

In summary, we observed that miRNA and the protein cargo of plasma EVs and their cellular origin were altered in patients with MHE. These changes would be responsible for the differential effects on CD4 T cell cultures. The addition to cultured CD4+ T lymphocytes from control subjects of EVs from MHE patients increased the expression of cytokines IL-17, IL-21, TNF-α, and CCL-20 and of the transcription factor related to Th follicular BCL6 compared to EVs from patients without MHE. These alterations are similar to those observed in MHE patients in a previous study, in which we showed that patients with MHE had higher plasma levels of IL-21, CCL20, TNF-α, and IL-22 compared to the healthy controls and patients without MHE, and higher levels of IL-1β only when compared to the healthy controls. On the other hand, both groups of cirrhotic patients had higher levels of IL-17 and lower levels of TGF-β compared to the healthy controls, with no differences between them [5]. These results suggest that EVs could play an important role in immune system modulation and in the induction of the immune changes observed in MHE patients.

## 4. Materials and Methods

### 4.1. Patients and Controls

Fifty patients with liver cirrhosis were consecutively recruited from the outpatient clinics at the Hospitals Clínico and Arnau de Vilanova of Valencia, Spain from January 2018 to May 2021. The diagnosis of cirrhosis was based on clinical, biochemical, and ultrasonographic data. Exclusion criteria were overt hepatic encephalopathy, recent (<6 months) alcohol intake, infection, recent (<6 weeks) antibiotic use or gastrointestinal bleeding, recent (<6 weeks) use of drugs affecting cognitive function, presence of hepatocellular carcinoma, or neurological or psychiatric disorder. Patients included in the study did not show fever or any clinical or biological sign of recent infection. Twenty-four healthy volunteers were also enrolled in the study once liver disease was discarded by clinical, analytical, and serological tests. All participants were included in the study after signing their written informed consent. The study protocols were approved by the Scientific and Ethical Committees of both hospitals. The procedures followed were in accordance with the ethical guidelines of the Declaration of Helsinki. The clinical and demographic variables of the participants are shown in Table 1.

### 4.2. Diagnosis of Minimal Hepatic Encephalopathy (MHE)

MHE was diagnosed in patients using the Psychometric Hepatic Encephalopathy Score (PHES) [40]. The scores were adjusted for age and education level using Spanish normality tables (www.redeh.org/TEST_phes.htm accessed on 27 May 2021). Patients were classified as having MHE when the score was ≤−4 points. Healthy volunteers also undertook PHES to discard any kind of cognitive impairment.

### 4.3. Extracellular Vesicle Isolation

EVs were isolated from frozen plasma by size-exclusion chromatography using the qEV 2/70 nm columns from IZON (Izon, Lyon, France). Plasma (5 mL) was thawed on ice and cell debris and large particles were removed by centrifuging the plasma at 1500 g for 10 min. Then, the samples were concentrated with Amicon^®^ Ultra-4 centrifugal filter 10 K devices (UFC801024 Millipore, Burlington, MA, USA) to 1 mL. Columns were equilibrated with 30 mL of 0.22-µm-filtered PBS. Plasma (1 mL) was added into the column and 0.22-µm-filtered PBS was added to the top of the column. Fractions 6 to 10 (500 µL each) were collected and concentrated in an Amicon^®^ Ultra 10 K device to 200 µL. A total of 6 uL was used for protein quantification by the colorimetric assay with bicinchoninic acid (BCA Protein Assay Kit Thermo Fisher Scientific Inc., Waltham, MA, USA) and the samples were stored at *−*80 °C.

### 4.4. Nanoparticle Tracking Analysis

The distribution profile, size, and quantity of EVs were assessed by nanoparticle tracking analysis (NTA) with a NanoSight NS300 system (Malvern, UK). A 1:1000 dilution of the EV samples was used for NTA. Five videos of 30 s were recorded at random points of each sample and were analyzed with NTA 3.2 Dev Build 3.2.16 software. According to the specifications and operational guide for the purification of EVs from IZON, an initial measurement of the EV concentration and protein purity of fractions (6–11) is recommended. Initially, all fractions from F0 to F40 were analyzed by Nanosight and by protein quantification. The elution fractions 6 to 10 were enriched with vesicles with a diameter of approximately 100–150 nm (Figure S1A,B). Coomassie blue staining in the fractions eluted (F1–F25 fractions) showed that plasma proteins began to be detected from the F11 fraction (Figure S2), and the EV concentration decreased from this fraction (see Figure S1A). The particle/protein ratio was calculated for the F6–F10 fractions, showing values of 13.3 × 10^10^ (F7), 8.7 × 10^10^ (F8), and 6.15 × 10^10^ (F9) (see Figure S1D), which indicates a good level of purity (>3 × 10^10^) [41].

Fractions 6–10 obtained from the IZON column were pooled and concentrated for each sample and analyzed in Nanosight. The particle mode size (particle size/mL) is provided by the Nanosight software. An example of the particle size distribution profiles measured by NTA in the three groups of study is shown in Figure 1D. The number of EVs was referred to the initial volume of plasma used to isolate EVs (5 mL) and expressed as number of particles/sample (Figure 1C).

### 4.5. Transmission Electron Microscopy

Electron microscopy was performed as described in [42]. Electron microscopy images of the EVs isolated are shown in Figure 1A.

### 4.6. Analysis of EVs Protein Cargo by Immunoblotting

Western blot analysis was conducted for different EVs markers as recommended by the International Society for Extracellular Vesicles (CD9, Flotillin-2 and Alix) [43] (Figure S3). To test the purity of the EVs, we analyzed the endoplasmic reticulum marker Calnexin by Western blot, which was absent in the population of EVs isolated (Figure S4).

Samples were subjected to electrophoresis and immunoblotting as in [44]. The primary antibodies used were against CD9, CD3, CD19, CD86, CD14, TNF-α, ADAM17, TNFR1, L1CAM, CD13, Flotillin-2, MHC I, MHC II, CD4, glutaminase, Alix, CD16, CD8, and glutamine synthetase. Antibody primary company, species and dilution used as well as the secondary antibodies are shown in Table S1. After scanning, the membrane band intensities were quantified using Alpha Imager 2200 version 3.1.3, and expressed as the percentage of mean intensity of the controls. The equivalent of 10 µg of EV protein was loaded on all Western blots and then calculated based on the control samples of each blot. GADPH and β-actin were used as the loading controls, depending on the molecular mass of the proteins analyzed. Representative blots with the corresponding loading control are shown in Figure S5.

### 4.7. Analysis of EVs TGF-β Cargo by ELISA

The TGF-β content in EVs was measured by a DuoSet ELISA Kit (R&D Systems) using 20 µg of total EV protein following the manufacturer’s instructions. Values were expressed as the percentage of the mean concentration of the controls.

### 4.8. Proteomic Analysis

The differential expression of proteins was analyzed at SCSIE Valencia University using EVs isolated from the plasma of 17 controls and 33 patients (18 without MHE and 15 with MHE). Selected samples of EVs were from subjects of similar age and similar gender proportions were maintained in the groups. An equivalent amount of all samples was pooled to build the spectral library from a 1D SDS-PAGE gel. The run corresponding to the library was cut and digested with sequencing grade trypsin, as described in [45]. Five microliters of each digested pool was loaded onto a trap column (LC Column, 3 µm C18-CL, 350 µm × 0.5 mm; Eksigent, Dublin, CA, USA) and desalted with 0.1% trifluoroacetic acid at 5 µL/min, 5 min. Peptides were loaded onto an analytical column (LC Column, 3 µm C18-CL, 0.075 × 150 mm, Eksigent) equilibrated in 5% acetonitrile, 0.1% formic acid. Peptide elution was carried out with a linear gradient of 7% to 40% B in A for 45 min (A: 0.1% formic acid; B: acetonitrile, 0.1% formic acid) at a flow rate of 300 nL/min. Peptides were analyzed in a mass spectrometer nanoESI qQTOF (6600 plus TripleTOF, ABSCIEX, Framingham, MA, USA) and in the Protein Pilot search engine (Sciex, Framingham, MA, USA) to generate a peak list. For individual SWATH analysis, 15 µg of protein was loaded in a 1D SDS-PAGE gel. The sample was digested with trypsin, as above. Peptides were analyzed in the mass spectrometer and the triple TOF was operated in swath mode, in which a 0.050 s TOF MS scan from 350–1250 *m*/*z* was performed, followed by 0.080 s product ion scans from 350–1250 *m*/*z* on the 37 defined windows (3.05 s/cycle). The Swath windows used were 15 Da window widths from 450 to 1000 Da, 37 windows. Individual SWATH injections were randomized and analyzed by Peak View 2.1.

### 4.9. miRNA Analysis

miRNA expression analysis was performed with plasma derived EVs from four control and nine patients (five without MHE and four with MHE) using the HTG EdgeSeq System (HTG Molecular Diagnostics, Inc., Tucson, AZ, USA) with the HTG EdgeSeq miRNA Whole Transcriptome Assay (miRNA WTA). The selected samples of EVs were from subjects of similar age and a similar gender proportion was maintained in the groups. This assay quantified the expression of 2083 human RNA transcripts (https://www.htgmolecular.com/assays/mirna-wta accessed on 29 October 2019). The same amount of EVs (3.6 × 10^9^ particles) from each patient was digested with proteinase K following the manufacturer’s instructions. miRNAs of interest were then selected by a protection nuclease assay (qNPA) in the HTG EdgeSeq processor using the miRNA Whole Transcriptome Assay (miRNA WTA) panel (HTG Molecular Diagnostics, Inc., Tucson, AZ, USA). The library prepared with the miRNA selected was amplified by PCR using adapters for the Next Seq 550 System sequencer (Illumina, San Diego, CA, USA). The normalization method CQN (conditional quantile normalization), implemented in the cqn R/Bioconductor package [46], was applied to remove the GC-content bias and correct for global distortions in the HTSeq data. Voom transformation [47] of the count-based HTSeq data was applied to estimate the mean-variance relationships. Linear modeling from the limma R/Bioconductor package [48] was performed to find differentially expressed miRNAs between groups. Target genes of miRNAs were found with a screening of five different databases (miRDB, miRTar, PITA, miRWalk, and TargetScan), keeping those pairs that were annotated in at least three databases and mandatory belonging to miRWalk and Target Scan. Differential expression information (inhibition) from miRNAs to target genes was transferred with the mdgsa R/Bioconductor package [49] and subsequent gene set analysis was applied using KEGG pathway database annotation [50].

### 4.10. CD4+ T Cell Culture and Treatment with EVs

CD4+ T cells were isolated from frozen PBMCs from healthy controls using the EasySep™ Human CD4+ T Cell Isolation Kit (StemCell Technologies, Vancouver, BC, Canada). The cell purity of isolated cells was checked by flow cytometry for CD3+ and CD4+ cells following the protocol as in the reference [5]; the purity of isolation was around 98% (Figure S6). CD4+ T cells (10^6^ cells/mL) were cultured in 48-well culture plates (500,000 cells/well) for 5 days in X-VIVO 20 medium containing 1% penicillin/streptomycin at 37 °C with 5% CO_2_. Cells were stimulated with plate-bound anti-CD3 and soluble anti-CD28 and treated with 10 µg of isolated EVs from the different types of subjects (control, without MHE or MHE) or PBS. Samples used to treat the CD4+ T cells were carefully selected based on: (1) age and gender of the subject from whom the sample was isolated; (2) that the protein charge analyzed in these samples was representative of the mean of the study group to which they belonged; and (3) that the protein concentration of the samples used in each individual experiment was similar. After 24 h, aliquots of the supernatant were collected; the remaining medium and cells were collected after 5 days of culture and stored at −80 °C.

#### 4.10.1. Determination of Cytokine Production by ELISA

The cytokine concentrations released in the culture medium were measured by DuoSet ELISA Kit (R&D Systems). The concentrations of TNF-α, IL-21, IL-22, IL-17, and TGF-β were measured at 24 h and 5 days; IL-1β and CCL20 only at 5 days.

#### 4.10.2. Analysis of Transcription Factors by Quantitative Real-Time PCR (qPCR)

The RNA of the cell culture pellet was extracted using the TRIzol RNA Isolation Reagent (Sigma Aldrich, Burlington, MA, USA). For the extraction details, see the Supplementary Materials. The cDNA was synthetized from RNA using a High-Capacity RNA-to-cDNA Kit and qPCR was performed using Taqman™ assays labeled with FAM and TaqMan™ Gene Expression Master Mix following the manufacturer’s instructions (all reagents from Applied Biosystems, Waltham, MA, USA). Taqman™ gene expression assays used: TBX21 (Hs00203436_m1), GATA3 (Hs00231122_m1), FOXP3 (Hs01085834_m1), RORC (Hs01076122_m1), AHR (Hs00907314_m1), and BCL6 (Hs00153368_m1). The ΔΔCt method used to determine the target expression referred to the non-EV condition using HPRT1 (Hs02800695_m1) as a normalizer.

### 4.11. Statistical Analysis

Values are given as the mean ± standard error of mean (SEM) unless otherwise specified. The D’Agostino and Pearson omnibus normality test was used to test the variable normality. Between-group differences were analyzed using univariate analysis of covariance (ANCOVA) with age included as a covariate, followed by a post hoc Tukey’s multiple comparisons test. For non-parametric variables, the Kruskal–Wallis test was performed, followed by Dunn’s multiple comparisons test. Outliers were identified using the ROUT method, with Q = 1% [51]. The Pearson correlation analysis was used for correlations between the inflammatory markers in EVs and activated lymphocyte populations. For correlations with the PHES, the Spearman correlations were used. Results were analyzed by GraphPad PRISM Version 8 and SPSS version 26.0 (SPSS Inc, Chicago, IL, USA). The probability level accepted for significance was *p* < 0.05.

## Figures and Tables

**Table 1:** The clinical and demographic variables of the participants.

	Controls (n = 24)	Patients without MHE (n = 27)	Patients with MHE (n = 23)
Gender (M/F)	11/13	19/8	18/5
Age (years) ^a^	59 ± 7	63 ± 6	64 ± 8
Etiology of cirrhosis			
Alcohol		9	11
HBV/HCV		0/6	2/4
HBV/HCV + alcohol		1	1
NASH		8	4
Others		3	1
Ascites		0	10
Child Pugh score (A/B/C)		25/2/0	16/7/0
MELD score ^a^		8.2 ± 2	9.3 ± 2.6
Ammonia (uM) ^a^	13 ± 8	15 ± 9	40 ± 27 ***^/α^
Platelet count (×10^9^/L)	241 ± 56	115 ± 54 ***	130 ± 68 ***
PHES ^a^	0.7 ± 1.0	0.4 ± 1.0	−7.1 ± 3.2 ***^/ααα^

^a^ Values are expressed as mean ± SD. MHE, minimal hepatic encephalopathy; M, male; F, female; HBV, hepatitis B virus; HCV, hepatitis C virus; NASH, non-alcoholic steatohepatitis; MELD, model end stage liver disease; PHES, Psychometric Hepatic Encephalopathy Score. Values significantly different from control are indicated by an asterisk (*) and from NMHE patients by α. (α: *p* < 0.05; ***/ααα: *p* < 0.001).

## Data Availability

The data are contained within the article and the supplementary materials. The proteomic and miRNAomic data are openly available from the Zenodo.org public repository at https://doi.org/10.5281/zenodo.7137964 (accessed on 3 October 2022).

## Supplementary Materials

The following supporting information can be downloaded at: https://www.mdpi.com/article/10.3390/ijms232012335/s1.
