# Supplementary material for: Plasma Extracellular Vesicles Play a Role in Immune System Modulation in Minimal Hepatic Encephalopathy
Source: Int J Mol Sci. 2022 Oct 15;23(20):12335. doi: 10.3390/ijms232012335 (PMC9604313; doi:10.3390/ijms232012335)
Supplement: Supplementary file 1 [file ijms-23-12335-s001.zip › ijms-1852510-supplementary.pdf]

**SUPPLEMENTARY MATERIAL**

# Plasma extracellular vesicles play a role in immune system modulation in minimal hepatic encephalopathy

Juan José Gallego, Alessandra Fiorillo, Franc Casanova-Ferrer, Amparo Urios, Mª Pilar Ballester, Lucia Durbán, Javier Megías, Teresa Rubio, Andrea Cabrera-Pastor, Desamparados Escudero-García, Vicente Felipo, Carmina Montoliu*

**Contents:**

**Supplementary methods:** RNA extraction from cell culture pellet

**Figure S1.** Nanosight analysis and protein quantification of F5 to F17 fractions eluted from plasma sample.

**Figure S2.** Protein detection by Coomassie blue staining in fractions eluted (F1-F25 fractions) from two plasma samples.

**Figure S3.** Representative bands, relative cargo and whole blot of extracellular vesicles markers.

**Figure S4.** Calnexin representative SDS-PAGE western blots.

**Figure S5.** Representative bands and relative extracellular vesicles protein cargo.

**Figure S6.** Purity of the $CD4^+$ T cell sample isolated using the EasySep™ Human $CD4^+$ T Cell Isolation Kit.

**Table S1.** Primary antibody and dilution used in analysis of EVs protein cargo by immunoblotting.

**Table S2.** Potential biological function of proteins differentially expressed in EVs from MHE assessed using Gene Ontology (GO) platform.

**Table S3.** Correlations between protein content of EVs, PHES score and activation of lymphocytes subpopulations

**Supplementary methods**

**RNA extraction from cell culture pellet**

The RNA of cell culture pellet was extracted using TRIzol RNA Isolation Reagent (Sigma Aldrich). Cells were homogenized with 300 µL of TRIzol RNA Isolation Reagent, mixture with chloroform and centrifuged 15 minutes at 13.000 g at 4ºC. Aqueous phase containing RNA was transferred to new tube and mixture with isopropyl alcohol to precipitate RNA, then centrifuged 15 minutes at 13.000 g at 4ºC and was washed with cold 75% ethanol. Finaly, RNA was resuspended in RNase-free water. Concentration and quality of RNA was checked using NanoDrop™ 2000 (Thermo Fisher).

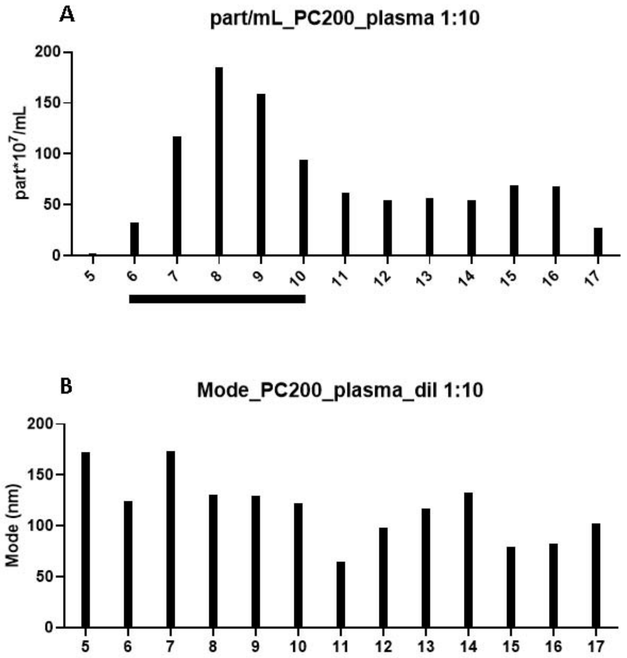


C

| PC200-PLASMA | Protein UG/UL |
|---|---|
| F1 | 0,000 |
| F2 | -0,002 |
| F3 | -0,002 |
| F4 | 0,000 |
| F5 | -0,002 |
| F6 | -0,001 |
| F7 | 0,009 |
| F8 | 0,020 |
| F9 | 0,026 |
| F10 | 0,044 |
| F11 | 0,088 |
| F12 | 0,160 |
| F13 | 0,329 |
| F14 | 1,089 |
| F15 | 1,673 |
| F16 | 2,269 |
| F17 | 2,594 |
| F18 | 2,922 |

D

| Fraction | Particles/mL(*$10^7$) | Protein (mg/mL) | Ratio Particles/Protein (*$10^{10}$) |
|---|---|---|---|
| 6 | 40 | -0,001 | |
| 7 | 120 | 0,009 | 13,3 |
| 8 | 175 | 0,02 | 8,7 |
| 9 | 160 | 0,026 | 6,15 |
| 10 | 100 | 0,044 | 2,2 |
| 11 | 60 | 0,088 | 0,6 |
| 12 | 50 | 0,16 | 0,3 |
| 13 | 55 | 0,329 | 0,16 |

**Figure S1. Nanosight analysis and protein quantification of F5 to F17 fractions eluted from plasma sample. A)** Fractions F6-F10 are enriched in EVs. **B)** Size (nm) of eluted fractions. F6 to F10 fractions contained EVs with 100-150 nm of size. **C)** Protein quantification in fractions. **D)** Particles/protein ratio in fractions F7-F13

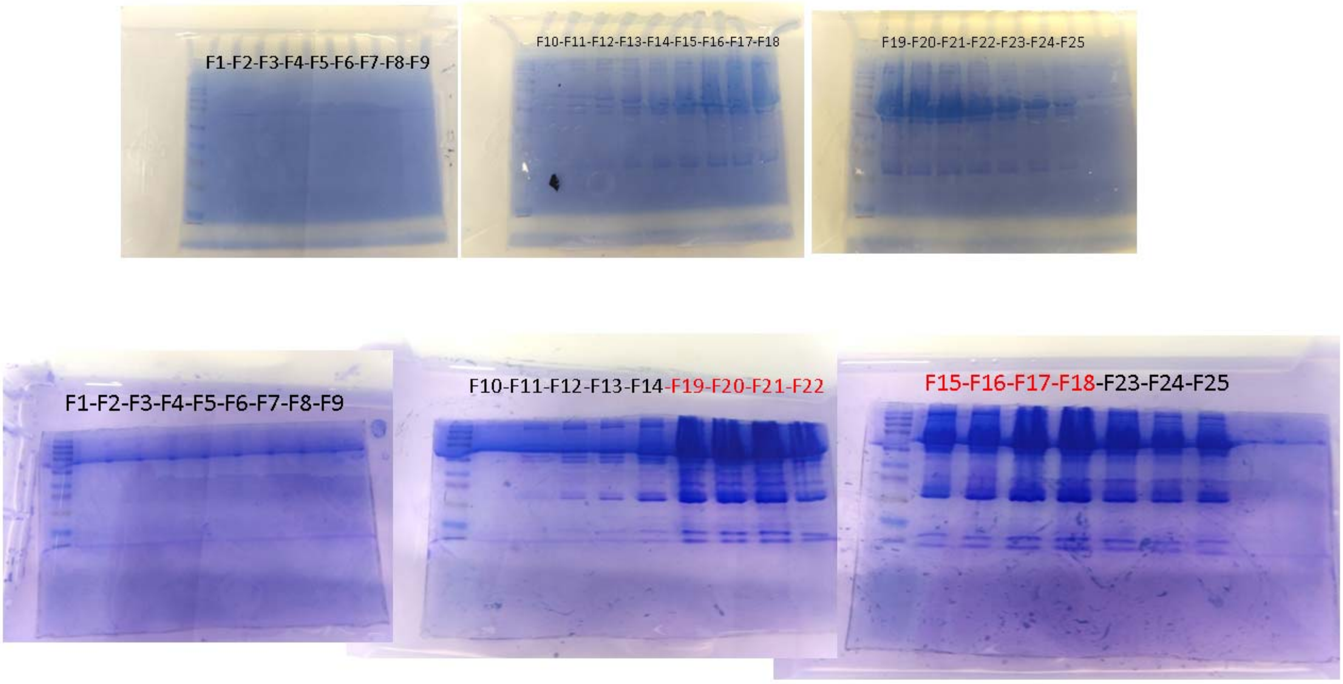


**Figure S2.** Protein detection by Coomassie blue staining in fractions eluted (F1-F25 fractions) from two plasma samples.

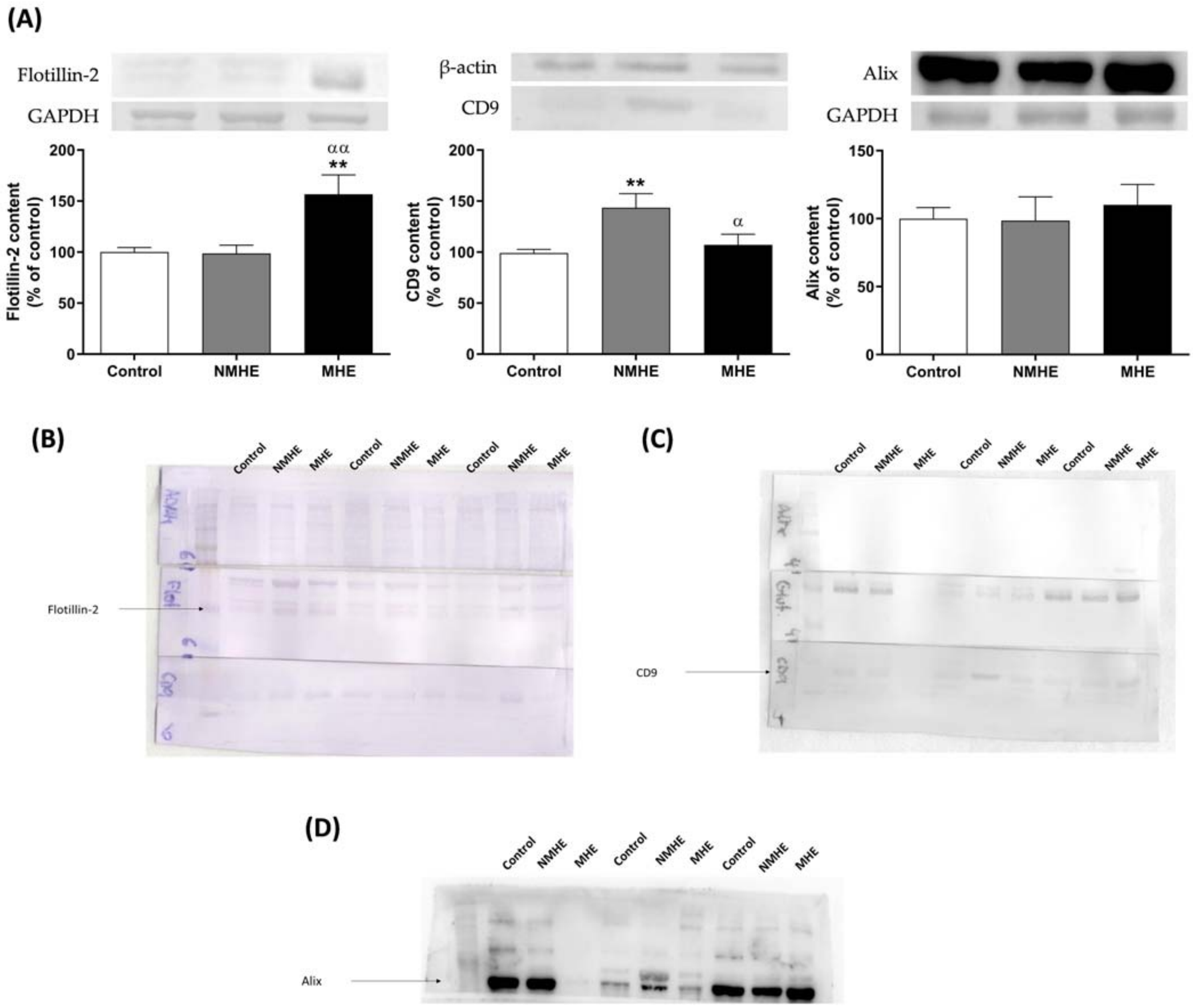


**Figure S3. Representative bands, relative cargo and whole blot of extracellular vesicles markers.** **(A)** Content of extracellular vesicle markers. (**B-D**) Whole blots of **(B)** flotillin-2, **(C)** CD9 and **(D)** Alix. Depending of molecular weight, β-actin or GAPDH were used as loading control. Values are expressed as percentage of control and are the mean ± SEM. Data were analyzed by univariate analysis of covariance (ANCOVA) with age included as a covariate, followed by Tukey post hoc test. NMHE, MHE, patients without and with minimal hepatic encephalopathy, respectively. Values significantly different from control are indicated by asterisk (*) and from NMHE patients by α. (α: $p < 0.05$; **/αα: $p < 0.01$).

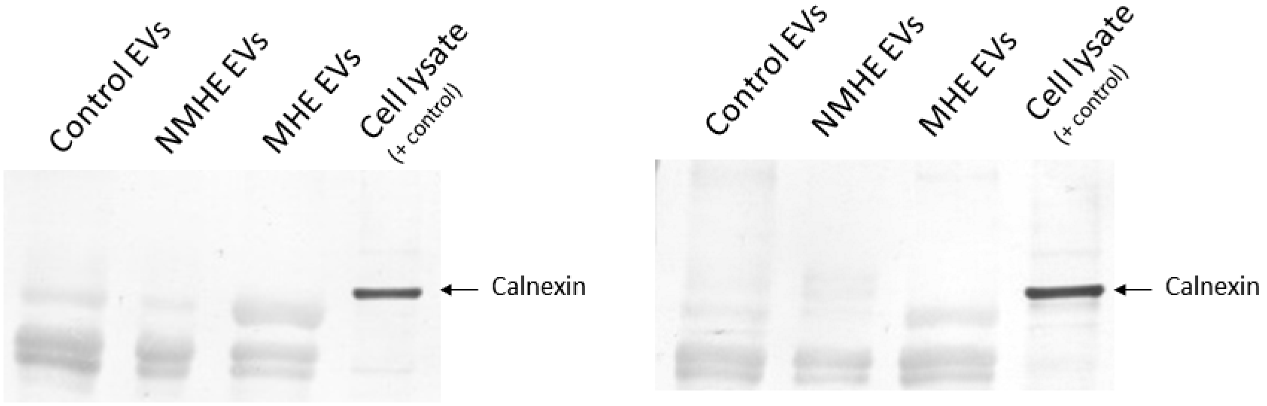


**Figure S4. Calnexin representative SDS-PAGE western blots.** Calnexin, marker of endoplasmic reticulum, was absent in EVs samples. Positive control used for calnexin was cell lysate of $CD4^{+}$ T cells.

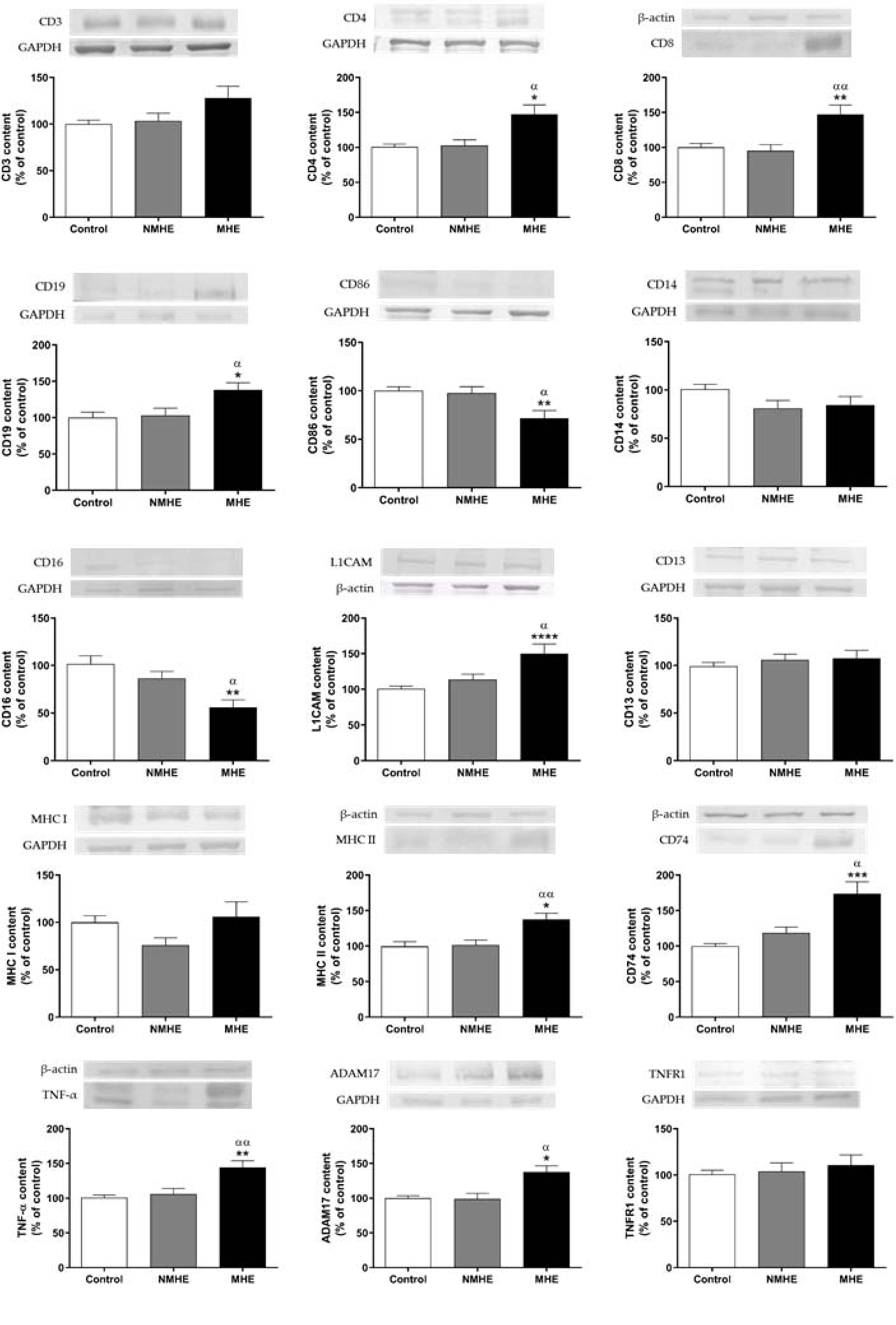

CD3
GAPDH
CD3 content (% of control)
0
50
100
150
Control
NMHE
MHE
CD4
GAPDH
CD4 content (% of control)
0
50
100
150
200
α
*
Control
NMHE
MHE
β-actin
CD8
CD8 content (% of control)
0
50
100
150
200
αα
**
Control
NMHE
MHE
CD19
GAPDH
CD19 content (% of control)
0
50
100
150
200
α
*
Control
NMHE
MHE
CD86
GAPDH
CD86 content (% of control)
0
50
100
150
α
**
Control
NMHE
MHE
CD14
GAPDH
CD14 content (% of control)
0
50
100
150
Control
NMHE
MHE
CD16
GAPDH
CD16 content (% of control)
0
50
100
150
α
**
Control
NMHE
MHE
L1CAM
β-actin
L1CAM content (% of control)
0
50
100
150
200
α
****
Control
NMHE
MHE
CD13
GAPDH
CD13 content (% of control)
0
50
100
150
Control
NMHE
MHE
MHC I
GAPDH
MHC I content (% of control)
0
50
100
150
Control
NMHE
MHE
β-actin
MHC II
MHC II content (% of control)
0
50
100
150
200
αα
*
Control
NMHE
MHE
β-actin
CD74
CD74 content (% of control)
0
50
100
150
200
α
***
Control
NMHE
MHE
β-actin
TNF-α
TNF-α content (% of control)
0
50
100
150
200
αα
**
Control
NMHE
MHE
ADAM17
GAPDH
ADAM17 content (% of control)
0
50
100
150
200
α
*
Control
NMHE
MHE
TNFR1
GAPDH
TNFR1 content (% of control)
0
50
100
150
Control
NMHE
MHE

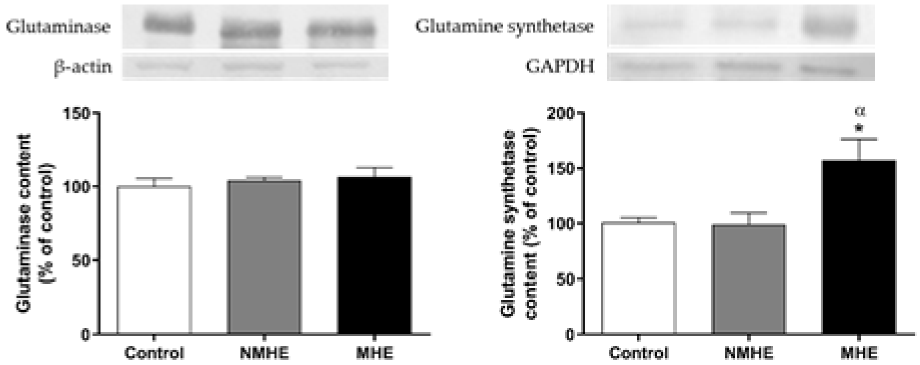


**Figure S5. Representative bands and relative extracellular vesicles protein cargo**. Content of markers related to immunity cells CD3, CD4, CD8, CD19, CD86, CD14 and CD16; markers related to CNS cells L1CAM and CD13; molecules implicated in activation of immune system and presenting antigens to T cells MHC I, MHC II and CD74; molecules related to inflammation environment TGF-β, TNF-α, ADAM17 and TNFR1; and enzymes related to ammonia metabolism, glutaminase and glutamine synthetase. Depending of molecular weight, β-actin or GAPDH were used as loading control. Values are expressed as percentage of control and are the mean ± SEM. Data were analyzed by univariate analysis of covariance (ANCOVA) with age included as a covariate, followed by Tukey post hoc test. NMHE, MHE, patients without and with minimal hepatic encephalopathy, respectively. Values significantly different from control are indicated by asterisk (*) and from NMHE patients by α. (*/α: $p < 0.05$; **/αα: $p < 0.01$; ***: $p < 0.001$; ****: $p < 0.0001$).

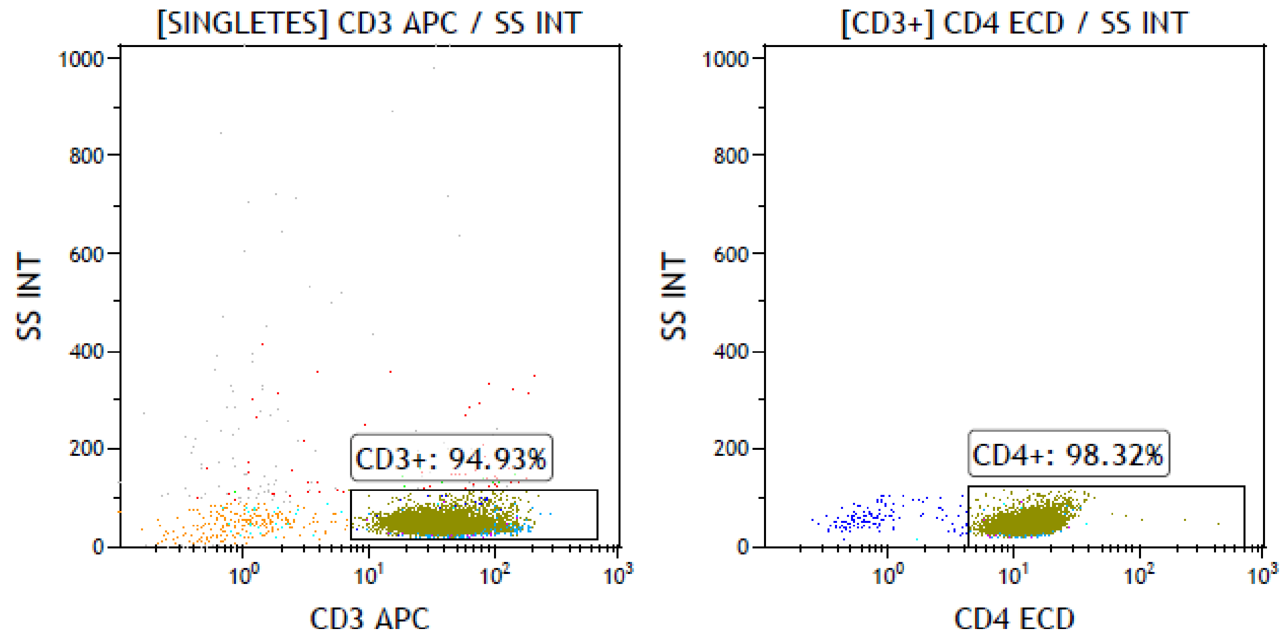


**Figure S6. Purity of the CD4⁺ T cell sample isolated using the EasySep™ Human CD4⁺ T Cell Isolation Kit.** After thawing the PBMC, the analysis of specific markers for CD4⁺ T cells was performed to determine the purity of the CD4⁺ sample following the protocol as in the reference [5]. The markers used were CD3 (total T cells) and CD4 (CD4⁺ T cells). CD4⁺ T cells were identified as positive for both markers.

**Table S1.** Primary antibody and dilution used in analysis of EVs protein cargo by immunoblotting.

| Protein | Primary antibody | | |
|---|---|---|---|
| | Species | Dilution | Company |
| CD9 | Rabbit (rb) | 1:500 | Sigma-Aldrich |
| Alix[a] | Rabbit (rb) | 1:1000 | Proteintech |
| Flotillin-2 | Rabbit (rb) | 1:500 | Invitrogen |
| TNF-α | Rabbit (rb) | 1:500 | Abcam |
| TNFR1 | Rabbit (rb) | 1:1000 | Abcam |
| ADAM17 | Rabbit (rb) | 1:250 | Abcam |
| Glutamine synthetase | Mouse (ms) | 1:5000 | Thermo Fisher |
| Glutaminase | Rabbit (rb) | 1:1000 | Novus Biological |
| MHC I | Rabbit (rb) | 1:1000 | Invitrogen |
| MHC II | Rabbit (rb) | 1:1000 | Invitrogen |
| CD74 | Mouse (ms) | 1:1000 | Abcam |
| L1CAM | Rabbit (rb) | 1:1000 | Bioss |
| CD13 | Rabbit (rb) | 1:500 | Bioss |
| CD3 | Rat (rat) | 1:500 | Abcam |
| CD4 | Rabbit (rb) | 1:1000 | Novus Biological |
| CD8 | Rabbit (rb) | 1:2000 | Thermo Fisher |
| CD19 | Rabbit (rb) | 1:1000 | Abcam |
| CD14 | Rabbit (rb) | 1:1000 | Abcam |
| CD16 | Rabbit (rb) | 1:100 | Santa Cruz |
| CD86 | Mouse (ms) | 1:200 | Abcam |
| Calnexin | Rabbit (rb) | 1:1000 | Novus Biological |
| β-actin | Mouse (ms) | 1:5000 | Abcam |
| GAPDH | Mouse (ms) | 1:15000 | Sigma-Aldrich |

Secondary antibodies used were Anti-rabbit, anti-mouse or anti-rat IgG conjugated with alkaline phosphatase (Sigma-Aldrich) at a dilution of 1:4000.

[a]For Alix protein, secondary antibody used was anti-rabbit IgG conjugated with horseradish peroxidase (Sigma-Aldrich) at a dilution of 1:4000.

**Table S2 (I).** Potential biological function of proteins differentially expressed in EVs from MHE assessed using (GO) platform

| GO biological process complete |
|---|
| **regulation of opsonization** |
| biological regulation |
| regulation of immune effector process |
| regulation of immune system process |
| **response to symbiotic bacterium** |
| response to symbiont |
| response to other organism |
| biological process involved in interspecies interaction between organisms |
| response to external biotic stimulus |
| response to biotic stimulus |
| response to stimulus |
| **negative regulation of complement activation, classical pathway** |
| negative regulation of humoral immune response mediated by circulating immunoglobulin |
| regulation of humoral immune response mediated by circulating immunoglobulin |
| regulation of immune response |
| regulation of response to stimulus |
| regulation of humoral immune response |
| negative regulation of humoral immune response |
| negative regulation of immunoglobulin mediated immune response |
| negative regulation of B cell mediated immunity |
| regulation of complement activation, classical pathway |
| regulation of complement activation |
| negative regulation of complement activation |
| **very-low-density lipoprotein particle assembly** |
| fibrinolysis |
| negative regulation of blood coagulation |
| negative regulation of coagulation |
| regulation of coagulation |
| negative regulation of multicellular organismal process |
| regulation of blood coagulation |
| regulation of wound healing |
| regulation of response to wounding |
| regulation of hemostasis |
| regulation of body fluid levels |
| regulation of biological quality |
| negative regulation of hemostasis |
| negative regulation of wound healing |
| negative regulation of response to wounding |
| **negative regulation of viral entry into host cell** |
| negative regulation of viral life cycle |
| **acute inflammatory response** |
| defense response |
| response to stress |

**Table S2 (II)**

| GO biological process complete |
|---|
| **complement activation, classical pathway** |
| humoral immune response mediated by circulating immunoglobulin |
| humoral immune response |
| immune response |
| immune system process |
| immunoglobulin mediated immune response |
| B cell mediated immunity |
| adaptive immune response based on somatic recombination of immune receptors built from immunoglobulin superfamily domains |
| adaptive immune response |
| lymphocyte mediated immunity |
| leukocyte mediated immunity |
| immune effector process |
| complement activation |
| activation of immune response |
| positive regulation of immune response |
| positive regulation of response to stimulus |
| positive regulation of immune system process |
| **renal system process** |
| **platelet degranulation** |
| **phagocytosis, engulfment** |
| plasma membrane invagination |
| membrane invagination |
| **negative regulation of endopeptidase activity** |
| regulation of endopeptidase activity |
| regulation of peptidase activity |
| regulation of hydrolase activity |
| regulation of catalytic activity |
| regulation of proteolysis |
| regulation of protein metabolic process |
| negative regulation of peptidase activity |
| negative regulation of proteolysis |
| negative regulation of protein metabolic process |
| negative regulation of hydrolase activity |
| negative regulation of catalytic activity |
| negative regulation of molecular function |
| **blood coagulation** |
| coagulation |
| hemostasis |
| wound healing |
| response to wounding |
| **innate immune response** |
| defense response to other organism |

**Table S3.** Correlations between protein content of EVs, PHES score and activation of lymphocytes subpopulations

| Protein in EVs | | PHES score[a] | Activated $CD3^+$ lymphocytes | Activated $CD8^+$ lymphocytes | Activated memory $CD4^+$ lymphocytes | Activated $CD8^+$ $CD28^-$ lymphocytes |
|---|---|---|---|---|---|---|
| TNF-α | r | -0.419 | 0.470 | 0.447 | 0.470 | 0.407 |
| | p | <0.01 | 0.015 | 0.02 | 0.015 | 0.04 |
| TGF-β | r | 0.297 | | -0.419 | -0.462 | -0.479 |
| | p | <0.05 | | 0.047 | 0.03 | 0.02 |
| MHCII | r | -0.280 | | | | |
| | p | <0.05 | | | | |

Correlation coeficient (r) and significance (p) for Pearson correlation analysis are shown. [a] For PHES score Spearman correlation analysis was used. Activation of lymphocytes subpopulations was measured as $CD69^+$ cells. EVs, extracelular vesicles; PHES, Psychometric Hepatic Encephalopathy Score.
